# Effects of Overload Current on the Ignition and Burning Hazards of Polyethylene-Insulated Wires

**DOI:** 10.3390/polym18050641

**Published:** 2026-03-05

**Authors:** Heran Song, Qingwen Lin, Zhurong Dong, Songfeng Liang, Ruichao Wei, Zhanyu Li, Shenshi Huang, Yiting Yan, Yang Li

**Affiliations:** 1 School of Automobile and Transportation Engineering, Shenzhen Polytechnic University, Shenzhen 518055, China; songheran@szpu.edu.cn (H.S.); ling@szpu.edu.cn (S.L.); richardwei@szpu.edu.cn (R.W.); autolzy@szpu.edu.cn (Z.L.); 2 School of Architectural Engineering, Shenzhen Polytechnic University, Shenzhen 518055, China; huangshenshi@szpu.edu.cn; 3 Forensic Science Institute, China People’s Police University, Langfang 065000, China; 2025909018@cppu.edu.cn (Y.Y.); liyang02@cppu.edu.cn (Y.L.)

**Keywords:** overload current, polyethylene-insulated wires, Ignition and burning, fire hazard, entropy-weight TOPSIS

## Abstract

To quantitatively elucidate the effects of overload current on the ignition and burning hazards of polyethylene-insulated wires, 2.5 mm^2^ polyethylene-insulated copper wires used commercially were tested in an electrical fire fault simulation system. Experiments were conducted to study the evolution of overloads, ignition, and burning. The entire process, from insulation smoking and ignition to sustained burning and final extinction driven by wire fusing, was recorded using synchronized digital and high-speed imaging. Video-based measurements were used to extract the following: smoking emission duration, ignition time, burning duration, maximum flame height, and segmented flame width. The results show that stable ignition and sustained burning occur when the overload current is greater than or equal to 180 A. As the current increases, ignition occurs earlier, while the smoking stage becomes shorter but exhibits nonmonotonic fluctuations. The burning duration shows a staged response. It first increases, then decreases toward a relatively stable level. This reflects the competition between enhanced Joule heating and accelerated wire melting and fusing. Maximum flame height and segmented flame width vary nonmonotonically with current, and the segmented flame width peaks at 200 A. A multi-indicator fire hazard evaluation framework was established and an entropy-weight TOPSIS method was applied to integrate the quantification and ranking. The overall fire hazard is greatest at 200 A. These findings provide experimental insight into overload-induced ignition and combustion behavior and contribute to a quantitative understanding of fire hazard evolution in overloaded electrical wires.

## 1. Introduction

Electrical fires commonly occur in energized wiring systems where ignition is initiated by abnormal heating or electrical discharges caused by faults rather than by external heat exposure. Previous studies suggest that ignition can be triggered by several failure pathways, such as overload operation and contact degradation, which increases local contact resistance. This generates localized Joule heating, which can evolve into arc faults or short circuits in some cases [1]. Despite the prevalence of fault-initiated fires, Babrauskas et al. [2,3,4] noted that the governing mechanisms and key parameters linking fault heat-source formation, localized heat accumulation, and the onset of sustained combustion remain insufficiently clarified. Among these pathways, overload is particularly insidious due to its long duration and cumulative nature. It often leads to a progressive temperature rise, insulation pyrolysis, and early-stage degradation that are difficult to detect in time. Therefore, systematic overload ignition and combustion experiments on polyethylene-insulated wires relevant to engineering applications are needed to quantify how overload current affects ignition timing, combustion persistence, and flame morphology. This will support the development of risk assessments and hazard criteria for overloads.

Scholars worldwide have conducted extensive research on the formation of heat sources, insulation degradation, and the behavior of ignition triggered by electrical circuit faults. Zhang et al. [5,6] conducted experimental and modeling studies on glow contact to characterize its temperature distribution and heat generation intensity. They found that localized increases in contact resistance and oxidation bridge formation can create sustained high-temperature heat sources. These sources can lead to the pyrolysis and carbonization of the insulating layer. These processes may further induce arcing or short-circuit faults. Li et al. [7] revealed that the ignition process caused by poor electrical contact exhibits a phased characteristic and provided threshold parameters related to contact power and arc power. Zhou et al. [8] quantified the variation of ignition time and ignition temperature of combustible materials with current and distance through alternating current arc experiments. Novak et al. [9] noted that electrical circuit failures under combined mechanical damage and overload conditions may undergo a prolonged, gradual evolution process. The aforementioned studies demonstrate from various perspectives that there exists a close coupling relationship between heat source formation, insulation degradation, and ignition behavior in electrical circuit failures.

Among various electrical circuit failure mechanisms, overload or overcurrent is considered one of the primary causes of wire insulation thermal failure and ignition due to its sustained action and tendency to accumulate during operation. Li et al. [10] demonstrated through experimental studies that wire insulation can break down under overload conditions, leading to repeated arcing events. They established a clear correlation between the time from overload onset to the first arc occurrence and the current magnitude. Subsequent numerical and experimental investigations further revealed the temperature distribution and breakdown locations in overloaded cables, while also identifying the threshold current range that triggers short circuits [11]. Under varying environmental and external field conditions, Fujita et al. [12] investigated ignition phenomena caused by short-term overcurrents in microgravity environments. Guo et al. [13,14] identified distinct ignition patterns under continuous overload conditions and analyzed the characteristics of ignition energy as a function of current. Jia et al. [15] examined the variation in spontaneous ignition delay of overloaded wires under crosswind conditions. Under conventional gravity conditions, Wang et al. [16] systematically characterized the combustion evolution process during overcurrent faults. Deng et al. [17] highlighted the crucial role of fuse-disconnect arcs in igniting overloaded wires. Gu et al. [18] analyzed overload ignition behavior and its controlling factors under low-pressure environments. Beyond ignition timing, overload currents also alter flame morphology and spatial scale characteristics post-ignition: Zhang et al. [19] observed a non-monotonic response in flame height and width with increasing current in experiments using energized polyethylene-insulated wires, with a marked decline occurring at higher current ranges; Tang et al. [20] demonstrated through heat transfer analysis that thermal feedback from the metal core to the insulation layer plays a significant role during combustion, with its relative contribution altering under high-current conditions; Zhao et al. [21] further indicated that both current magnitude and wire orientation/inclination jointly influence the combustion process’s response characteristics to current.

Although existing research has provided significant insights into overload-induced ignition and combustion of energized wires, most previous studies have concentrated on ignition thresholds or flame spread behavior within relatively limited current ranges. In contrast, systematic quantification of post-ignition burning persistence, flame spatial evolution, and their interaction with conductor softening and fusing under higher overload currents remains insufficient. In particular, the potential non-monotonic hazard response arising from the competition between intensified Joule heating and accelerated metal-core failure has not been explicitly identified or experimentally clarified in earlier studies.

To address this gap, the present work focuses on commercially used 2.5 mm^2^ flame-retardant PE-insulated copper wires and investigates a stable-ignition high-overload regime (180–240 A). By extracting multi-scale temporal and spatial indicators—including ignition time, burning duration, maximum flame height, and segmented flame width—this study demonstrates that fire hazard does not increase monotonically with current but instead reaches a peak under moderate overload conditions due to the competition between intensified Joule heating and accelerated conductor failure. Because these indicators may exhibit inconsistent or even opposite trends with increasing current, hazard evaluation based on a single parameter can be misleading. Therefore, the Entropy Weight–TOPSIS method is adopted to determine objective weights based on data dispersion and to establish a unified ranking framework by evaluating the relative closeness to an ideal hazard state. Compared with subjective weighting or single-index approaches, this method reduces bias and is particularly suitable for multi-indicator experimental systems characterized by coupled thermal–electrical processes.

## 2. Experiment

### 2.1. Materials

This study selected flame-retardant polyethylene-insulated copper wire as the experimental subject. The wire has a cross-sectional area of 2.5 mm^2^ and a rated current of 20 A. This specification is commonly used in low-voltage distribution circuits for buildings, indoor fixed wiring, electrical systems in public buildings, and power supply branches in densely populated areas, making it highly representative of engineering applications. To meet fire safety requirements, aluminum hydroxide (Al(OH)_3_) is used as a halogen-free flame retardant in the polyethylene insulation. It acts through endothermic decomposition and water release at elevated temperatures, and is incorporated into the polyethylene matrix by industrial melt blending followed by extrusion coating. Key specifications are listed in Table 1.

### 2.2. Method

**Figure 1:**
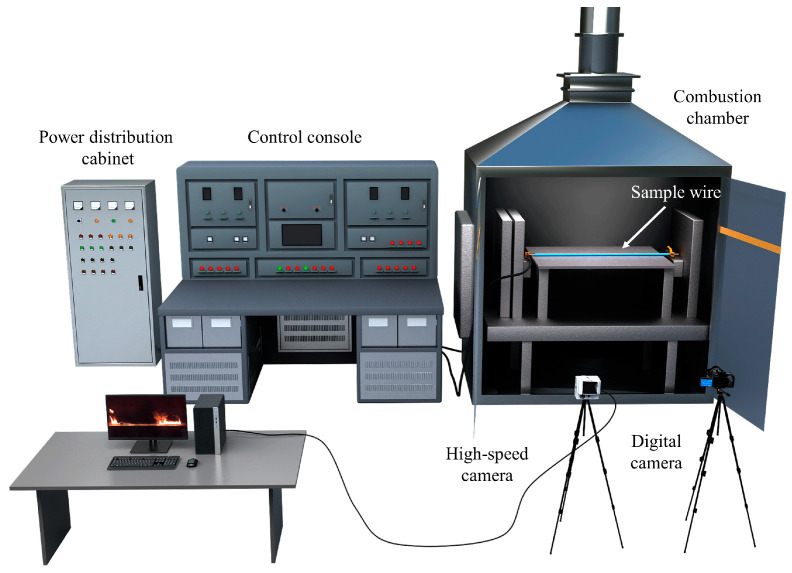
Experimental system for overloaded wire ignition and combustion.

As shown in Figure 1, the overload test system primarily consists of an electrical fire fault simulation device, a combustion chamber, a specimen fixation structure, and an image acquisition system. The electrical fire fault simulation device creates the wire overload condition by applying a stable, adjustable direct current (DC) overload current to electrical circuits. During the experiment, the device’s current output capability, ranging from 0 to 300 A, was used to apply overload loading to the specimen wires. The current control resolution of the device is 0.1 A. A DC power supply with low ripple characteristics was used to ensure waveform stability during testing. Prior to experiments, the output current was calibrated using a precision digital ammeter (Fujian Shunchang Hongrun Precision Instruments Co., Ltd., Nanping, China) (accuracy ±0.5% reading), and consistency between the preset and measured values was verified within the allowable deviation range. During each test, the current was continuously monitored to confirm stable operation throughout the overload process.

The specimen wire is positioned horizontally within the combustion chamber and is connected to the power circuit via copper clamping posts on either end. Axial tension is applied to keep the wire horizontal and taut and to maintain good electrical contact throughout the entire test. To simulate typical boundary conditions when wires are laid close to the ground in engineering applications, a support platform with ceramic tiles is installed beneath the wire. During the experiment, the effective energized length of the wire is 800 mm. The total specimen length is 840 mm, with approximately 20 mm of insulation stripped from each end before securing it to the wire clamping frame.

The current loading range was set to 100–240 A in 10 A increments. Three parallel experiments were conducted for each current condition. For each current level, the reported results are presented as mean values of the three repeated tests. Preliminary test results showed that, when the overload current was 180 A or greater, the specimens exhibited stable ignition and combustion phenomena. Therefore, this study’s statistical analysis and comprehensive evaluation of fire hazard indicators primarily focused on the 180–240 A range. The upper limit of 240 A was selected to characterize high-intensity electrical heating conditions in low-voltage wiring under severe overload. This selection aligns with engineering knowledge from electrical fire investigations regarding abnormal currents that cause thermal insulation failure [22].

To obtain multiscale information on the ignition and combustion processes of wires, digital and high-speed cameras were deployed simultaneously during the experiment. The digital cameras recorded the complete evolution from energization to flame extinction, including insulation smoking and ignition initiation. This data enabled the extraction of smoking emission duration ($t_{smoke}$), ignition time ($t_{ign}$), and burning duration ($t_{burn}$). The high-speed camera captured transient details of flame morphology during the early combustion stage and measured maximum flame height ($H_{\max}$) and maximum segmented flame width ($W_{\max,seg}$).

## 3. Ignition and Combustion Evolution of Overloaded Polyethylene Wires

**Figure 2:**
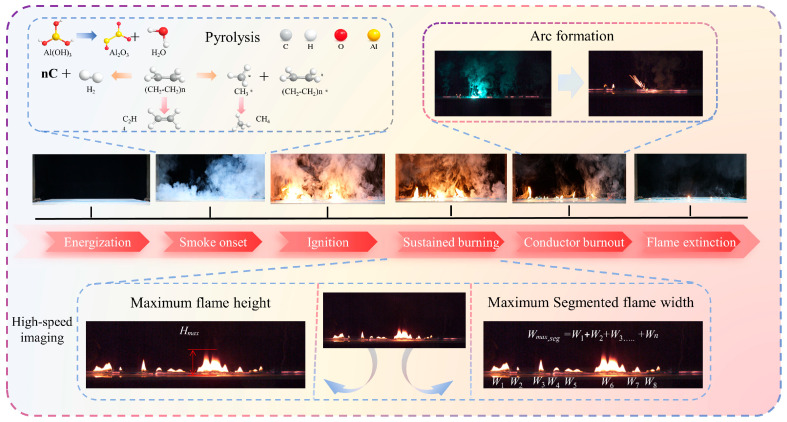
Typical fire development process of polyethylene-insulated wires under overcurrent conditions.

Under overload conditions, polyethylene wires undergo a distinct ignition and combustion process, as illustrated by the temporal evolution path in Figure 2. During the initial energization phase, electrical energy within the metal core is converted into thermal energy via the Joule effect. This thermal energy is then transferred radially toward the insulation layer as an internal heat source. The Joule heating power per unit length of the metal core can be expressed as

(1) $${\dot{q}}_{J}^{\prime }=I^{2}R^{\prime }$$

where I represents the applied current and $R^{\prime }$ denotes the unit length resistance of the metallic core. At this stage, visible combustion has not yet occurred, and the temperature evolution of the wire is primarily governed by the energy balance between internal electrical heating and external heat dissipation. Treating the wire as a unit-length control volume, the preheating process can be approximated as follows:

(2) $$m^{\prime }c_{p}\frac{d\bar{T}}{dt}={\dot{q}}_{J}^{\prime }-{\dot{q}}_{\mathrm{loss}}^{\prime }$$

This formulation represents a lumped-parameter approximation that neglects axial temperature gradients and assumes spatially averaged heat transfer. In the equation, $m^{\prime }$ represents mass per unit length, $c_{p}$ denotes equivalent specific heat capacity, $\bar{T}$ indicates representative average temperature, and ${\dot{q}}_{\mathrm{loss}}^{\prime }$ represents heat dissipation per unit length caused by surface convection and radiation. As the overload current increases, ${\dot{q}}_{J}^{\prime }$ rises significantly according to the $I^{2}$ law, causing the polyethylene insulation material’s temperature to enter the pyrolysis initiation range more rapidly. This trend is consistent with the experimentally observed monotonic reduction in ignition time with increasing current (Section 4.2), supporting the physical validity of the simplified preheating model.

When the temperature of the insulation layer reaches the pyrolysis initiation range of the insulating material, the flame-retardant aluminum hydroxide undergoes thermal dehydration, releasing water vapor and forming aluminum oxide. This process exhibits certain endothermic and diluting effects. During polyethylene pyrolysis, primarily saturated and unsaturated hydrocarbon gases and their cracking products are generated, including small-molecule hydrocarbons such as methane, ethylene, and ethane. These pyrolysis products are highly flammable and accumulate on the wire surface and in the surrounding space, providing a gaseous fuel source for subsequent ignition. Due to variations in axial temperature distribution and pyrolysis intensity along the wire, the release of pyrolysis gases is spatially non-uniform. This creates the conditions for segmented axial flame distribution during the combustion phase.

As the overload current persists, the heat generated within the wire intensifies. When the temperature of a localized area of the wire surface meets ignition conditions alongside a combustible gas concentration, the pyrolysis products in that region ignite, forming a stable, localized flame and initiating the fire event. After ignition, the combustion process transitions from being solely driven by electrical heating to a coupled process involving electrical heating and thermal feedback from the flame. The energy balance can be summarized as follows [23]:

(3) $$m^{\prime }c_{p}\frac{d\bar{T}}{dt}={\dot{q}}_{J}^{\prime }+{\dot{q}}_{f}^{\prime }-{\dot{q}}_{\mathrm{loss}}^{\prime }-{\dot{q}}_{\mathrm{py}}^{\prime }$$

The term ${\dot{q}}_{f}^{\prime }$ represents the convective and radiative heat feedback from the flame to the wire and the unburned insulation layer. The ${\dot{q}}_{\mathrm{py}}^{\prime }$ term denotes the equivalent energy consumption term that corresponds to the pyrolysis process. The coupled energy balance framework explains the staged variation in burning duration observed in Section 4.3, where moderate currents enhance flame feedback while higher currents accelerate conductor fusing and effectively remove the Joule heating term. During this stage, due to irregularities in axial temperature distribution, variations in pyrolysis release rates, and changes in local heat dissipation conditions, the flame does not propagate continuously along the wire. Instead, it forms several relatively independent combustion segments along the axial direction, exhibiting segmented combustion characteristics. The spatial position, duration, and scale of each combustion segment evolve over time and may be accompanied by local extinction and reignition phenomena.

During the sustained combustion phase, the thermal feedback effect of the flame intensifies and certain combustion segments gradually expand and interact with one another. The flame’s overall height increases while the lateral expansion of each segment varies significantly, resulting in a non-uniform axial distribution of flame width. As combustion progresses, a large-scale, high-temperature zone forms around the wire. Within the segmented combustion zones, the flame geometry reaches its maximum dimensions, which has a significant thermal radiation effect on the surrounding environment.

When exposed to high temperatures for an extended period of time, the metal core softens and melts. It gradually fractures under the combined effects of gravity and thermal stress, which interrupts the electrical current path. Following the interruption, the input of Joule heating ceases rapidly. The segmented combustion zones lose their internal heat source, causing the flames to progressively weaken and eventually extinguish. This terminates the overload ignition and combustion process [21].

Based on the typical evolution process shown in Figure 2 and considering the segmented combustion characteristics of flames along the wire axis, this paper defines and extracts key parameters for characterizing fire hazards. These parameters include $t_{smoke}$, $t_{ign}$, $t_{burn}$, $H_{\max}$, and $W_{\max,seg}$.

## 4. Results and Discussion

### 4.1. Smoke Emission Duration

**Figure 3:**
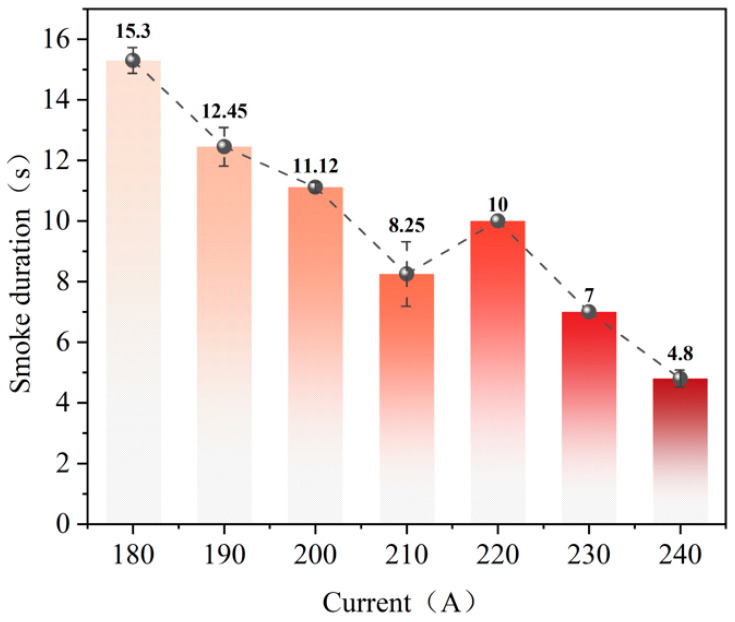
Smoke emission duration of polyethylene wire insulation under overload current.

Figure 3 shows how the duration of smoke emission from polyethylene wire insulation varies with current under different overload conditions. Within the 180–240 A range, the duration generally decreases as the current increases; however, a certain degree of non-monotonic variation is observed in the medium current range.

The smoking emission duration was longest at a current of 180 A, averaging approximately 15.3 s. As the current increased to 190 and 200 A, the smoke emission duration shortened to 12.45 and 11.12 s, respectively. This suggests that increased Joule heating from the metal core accelerated the insulation’s transition to the pyrolysis zone, hastening the pyrolysis reaction and shortening the smoke emission phase [24,25]. When the current increased to 210 A, the smoke emission duration decreased to approximately 8.25 s. This suggests that, at higher electrical heating intensities, the transition of the insulation material from pyrolysis to ignition is significantly accelerated. At 220 A, the smoke emission duration increased slightly to approximately 10 s, suggesting that, at this current level, the ignition trigger process does not advance monotonically with increasing current. These results suggest that the pyrolysis behavior of insulating materials does not strictly monotonically respond to current and that its variation may be related to the axial thermal state distribution along the wire [1]. At higher currents (230–240 A), smoke emission duration shortened significantly, decreasing to 7 and 4.8 s, respectively. At these currents, the Joule heat input per unit length increased markedly, causing the insulation temperature to rise rapidly. This compressed the pyrolysis phase, leading to a swift ignition process.

### 4.2. Ignition Time

**Figure 4:**
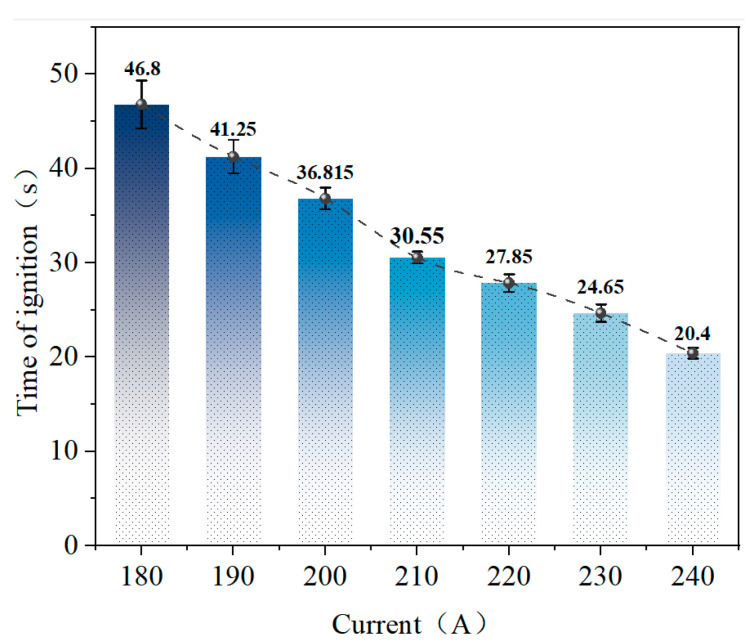
Ignition time of polyethylene wires under overload current.

Figure 4 illustrates the ignition time of polyethylene wires under various overload current conditions. As the overload current increases, the ignition time significantly shortens.

At an overload current of 180 A, the ignition time was relatively long at approximately 47 s, indicating that under conditions of limited Joule heating input, the wire required prolonged preheating and pyrolysis before igniting [26,27]. As the overload current increases, the ignition time decreases significantly, indicating that increased electrical heating intensity accelerates the temperature rise of the insulation material and the progression of pyrolysis. When the overload current rises to 240 A, the ignition time decreases to approximately 20 s, as the joule heat input per unit length increases markedly. This causes the insulation layer to rapidly enter the pyrolysis and ignition stages, substantially compressing the ignition delay.

### 4.3. Burning Duration

**Figure 5:**
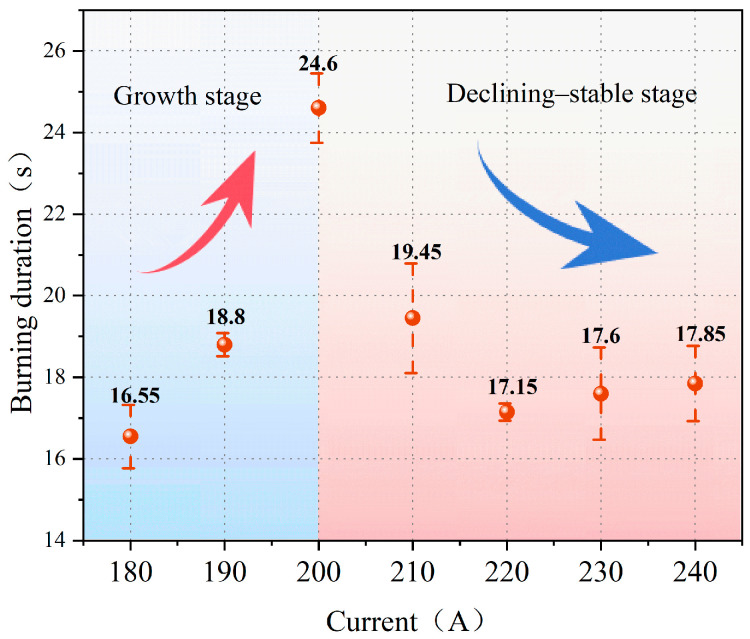
Burning duration of polyethylene wires under overload current.

Figure 5 illustrates the burning duration of polyethylene wires under various overload current conditions. The burning duration exhibits distinct phase characteristics with varying currents. It can be broadly divided into an initial growth phase at low currents, followed by a reduction phase and stabilization at high currents.

At lower overload current conditions, the burning duration gradually increases as the current rises. At 180 A, the burning duration is relatively short. When the current increases to 190 A, the burning duration extends and reaches its maximum at 200 A, indicating that the joule heat input per unit length of the metal core intensifies as the overload current increases. This process facilitates sustained pyrolysis and open-flame combustion of the insulating material for a longer period of time, thereby prolonging the burning duration.

When the overload current increases to 210 A or more, the burning duration no longer increases; instead, it decreases significantly and stabilizes. Although the Joule heating input continues to intensify at this point, the high temperatures accelerate the softening and melting of the metal core significantly. This causes the wire to break earlier, interrupting the current path and limiting the duration of the combustion process [17]. Gradually, the burning duration shifts from being dominated by enhanced Joule heating input to being controlled by metal core failure, which manifests as a reduction and stabilization of the burning duration.

### 4.4. Flame Characteristics

#### 4.4.1. Maximum Flame Height

**Figure 6:**
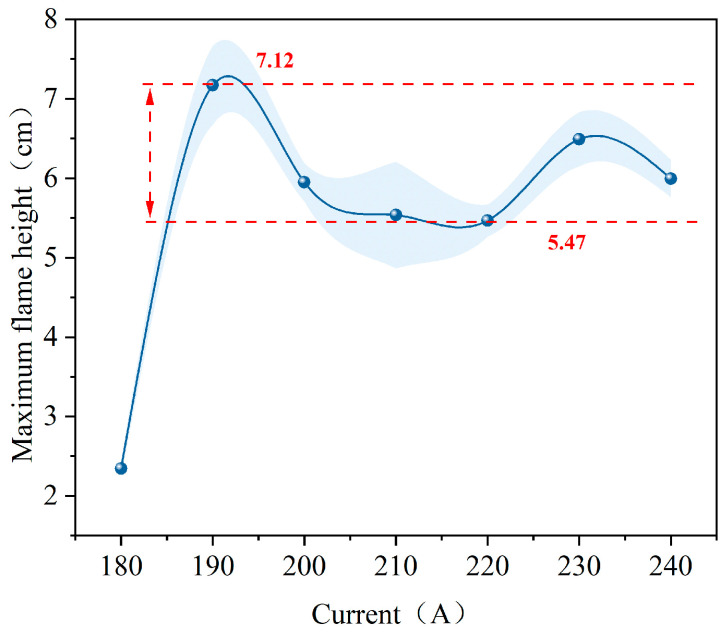
Maximum flame height of polyethylene wire under overload current.

Figure 6 illustrates the variation in maximum flame height for polyethylene wires under various overload current conditions. Overall, the maximum flame height exhibits a distinct non-monotonic trend with respect to current. It rises rapidly in the low-current range, then declines and stabilizes at a relatively constant level in the medium-current range. Finally, it fluctuates again in the high-current range.

At 180 amperes, the maximum flame height remained relatively low. As the current increased to 190 A, the maximum flame height increased significantly, reaching a higher level. Within the 190–240 A range, it fluctuated, generally falling within the range of approximately 5.47–7.12 cm. This suggests that, during the post-ignition phase, the vertical scale of the flame does not monotonically increase with current, but rather exhibits constrained growth characteristics across a broad range of overload currents. Under higher overload current conditions, the flame morphology is influenced by processes such as segmented combustion along the wire axis and the softening, melting, and fracturing of the metal core, which interrupts the current supply, despite the continuous increase in Joule heating input. These factors cause the maximum flame height to fluctuate within a certain range, preventing it from developing into larger values [28,29].

#### 4.4.2. Maximum Segmented Flame Width

**Figure 7:**
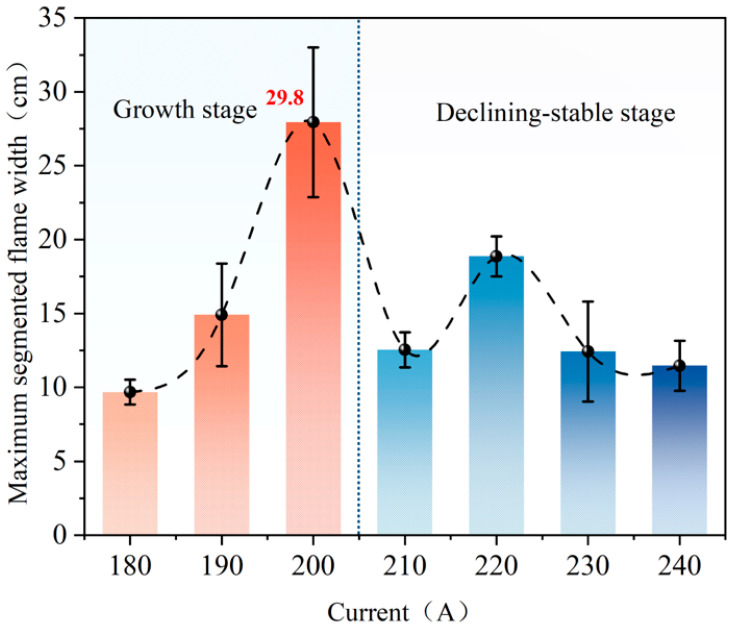
Maximum segmented flame width in polyethylene wires under overload current.

Figure 7 illustrates the variation in maximum segmented flame width for polyethylene wires under various overload current conditions. The maximum segmented flame width clearly exhibits distinct phase characteristics with current changes. It can be divided into a growth zone at low currents and a stable reduction zone at high currents. To characterize the flame’s lateral expansion capability under segmented combustion conditions, this paper denotes the widths of flame segments separated axially along the wire at the same instant as *W*_1_, *W*_2_, …, *W*_n_. The total segmented flame width at that instant is defined as

(4) $$W_{\mathrm{seg}}=\sum_{i=1}^{n}W_{i}$$

The maximum value of $W_{\mathrm{seg}}$ during combustion is defined as the maximum width of the flame segment $W_{\max,\mathrm{seg}}$. Here, $W_{i}$ is measured as the projected length of each independent flame segment along the wire axis in the calibrated image.

Under lower overload current conditions, the maximum width of the segmented flame increases significantly as the current rises. It is smaller at 180 A, increases markedly at 190 A, and reaches its maximum at 200 A, which is approximately 29.8 cm. This indicates that, as the joule heat input per unit length of the metal core intensifies, the thermal decomposition of the insulation layer becomes more pronounced. This leads to more extensive lateral expansion of the segmented combustion zone, consequently driving a rapid increase in maximum width.

As the overload current increases beyond 210 A, the maximum width of the segmented flame significantly declines and remains lower within the higher current range. A degree of recovery is observed near 220 A; however, the overall trend remains downward and stabilizes. This phase indicates that, despite continued increases in joule heating input, the accelerated softening, melting, and fracturing of the metal core lead to easier current interruption at higher overload currents. This limits the sustained development of segmented combustion zones and weakens the flame’s lateral expansion capability. As a result, the maximum width reduces and stabilizes.

## 5. Comprehensive Evaluation of Fire Hazards Under Overcurrent Conditions

The preceding section systematically analyzed the ignition and combustion behavior of polyethylene wires under different overload current conditions. The analysis focused on aspects such as insulation, smoke generation, ignition, combustion persistence, and flame morphology. The results indicate that individual metrics exhibit varying and even contradictory trends with changes in current, making it difficult for any single parameter to reflect the overall fire hazard level comprehensively. Thus, this study introduces the Entropy Weight–TOPSIS method, which performs a comprehensive calculation and ranking analysis of multi-indicator fire hazards. This method enables a quantitative comparison of fire hazards under different overload current conditions [30,31,32].

Select $t_{smoke}$, $t_{ign}$, $t_{burn}$, $H_{\max}$, and $W_{\max,seg}$ as fire hazard evaluation indicators. Using current condition (180–240 A) as the evaluation target, construct the original decision matrix $X=[x_{ij}]$. Experimental data for each indicator are summarized in Table 2.

Due to differing physical units and inconsistent hazard indicators, the raw data first undergoes directional unification and dimensionless processing to obtain the normalized matrix $R=[r_{ij}]$. For positive indicators $\left\{t_{\mathrm{burn}},\;H_{\max},\;W_{\max,\mathrm{seg}}\right\}$ where higher values indicate greater fire risk, the following approach is adopted:

(5) $$r_{ij}=\frac{x_{ij}-\min(x_{i})}{\max(x_{i})-\min(x_{i})}$$

For the inverse indicator $\left\{t_{smoke},\;t_{ign}\right\}$, where smaller values indicate higher fire risk, the following approach is adopted:

(6) $$r_{ij}=\frac{\max(x_{i})-x_{ij}}{\max(x_{i})-\min(x_{i})}$$

This yields $r_{ij}\in [0,1]$, where higher values indicate greater fire risk. Based on this, the entropy weight method is employed to determine the objective weights of each evaluation indicator. Let


(7) $$p_{ij}=\frac{r_{ij}}{\sum_{j=1}^{n}r_{ij}},\;\;\;(n=7)$$

Then the information entropy of the i indicator is:

(8) $$e_{i}=-k\sum_{j=1}^{n}p_{ij}\ln\left(p_{ij}\right),\;\;\;k=\frac{1}{\ln n}$$

Its information utility (differential value) is


(9) $$d_{i}=1-e_{i}$$

The corresponding entropy weight is


(10) $$w_{i}=\frac{d_{i}}{\sum_{i=1}^{m}d_{i}},\;\;\;(m=5)$$

The weights for each indicator calculated based on the data in Table 2 are shown in Table 3.

After obtaining the weights, construct the weighted normalized matrix $V=[v_{ij}]$, where


(11) $$v_{ij}=w_{i}r_{ij}$$

The TOPSIS method defines positive ideal solutions and negative ideal solutions as follows:

(12) $$v^{+}=\left\{\max\left(v_{ij}\right)\right\},\;\;\;v^{-}=\left\{\min\left(v_{ij}\right)\right\}$$

Calculate the Euclidean distance from each overload current condition to the positive and negative ideal solutions:

(13) $$S_{j}^{+}=\sqrt{\sum_{i=1}^{m}{\left(v_{ij}-v_{i}^{+}\right)}^{2}},\;\;\;S_{j}^{-}=\sqrt{\sum_{i=1}^{m}{\left(v_{ij}-v_{i}^{-}\right)}^{2}}$$

Based on this definition, the proximity index (comprehensive fire hazard index) is calculated as follows:

(14) $$C_{j}=\frac{S_{j}^{-}}{S_{j}^{+}+S_{j}^{-}},\;\;\;0\leq C_{j}\leq 1$$

**Figure 8:**
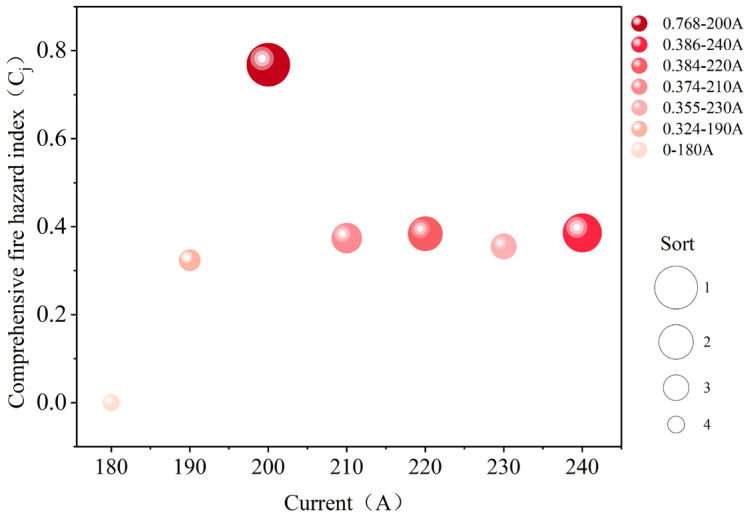
Comprehensive fire hazard index and ranking under different overload current conditions (TOPSIS Results).

The higher the value of $C_{j}$, the greater the fire hazard under that current condition. Based on the aforementioned process, calculations were performed for each current operating condition to obtain the proximity factor $C_{j}$ and rank them accordingly. The results are shown in Figure 8.

Figure 8 shows that the fire hazard is highest at 200 A and decreases as the current increases to 240 A, 220 A, 210 A, and 230 A. The overall hazard is lowest at 180 A. This indicates that the fire hazard does not increase monotonically with current, but rather peaks under moderate overload conditions. Within this current range, ignition time is significantly shorter, burning duration remains elevated, and maximum flame height and segmented maximum width are relatively large. Multiple hazard indicators overlap temporally and spatially, thereby elevating the overall fire hazard. In contrast, at higher overload currents, although ignition occurs earlier, combustion persistence and the spatial scale of flames are constrained by the rapid softening and fracturing of the metal core. Consequently, the overall fire hazard does not continue to increase.

The ranking result obtained from the Entropy Weight–TOPSIS method is physically consistent and statistically robust rather than being solely a mathematical outcome. At 200 A, ignition time is markedly reduced while burning duration and segmented flame width remain relatively high, indicating a balance between intensified Joule heating and delayed conductor fusing. At higher currents (≥210 A), although ignition occurs earlier, accelerated fusing truncates flame development. Therefore, the identification of 200 A as the most hazardous condition reflects the coupled physical mechanisms observed experimentally rather than merely data dispersion.

Regarding reliability, the method is deterministic for a given dataset, and uncertainty primarily originates from experimental variability. Based on three repeated tests per current level, the variation of the comprehensive hazard index remained within the dispersion range of the measured indicators and did not alter the overall ranking trend, with 200 A consistently identified as the highest-hazard condition. These results demonstrate the stability of the proposed evaluation framework under the present experimental conditions.

## 6. Conclusions

This study conducted ignition and combustion experiments on 2.5 mm^2^ flame-retardant, polyethylene-insulated, copper wires under overload currents ranging from 180 to 240 A, systematically characterizing the entire process, from preheating and insulation smoke emission to ignition, sustained combustion, and final extinction. A quantitative analysis was performed on the evolution of ignition timing, combustion persistence, and flame morphology with respect to current. The key findings are as follows:

(1) Under overload conditions, the ignition and combustion processes of polyethylene-insulated wires exhibit distinct, phased evolutionary characteristics. The wire undergoes preheating and insulation smoking when exposed to sustained Joule heating. Once ignition conditions are met in a localized area, combustion begins. After ignition, the flame propagates along the wire’s axis with segmented combustion characteristics. The flame ultimately extinguishes when the current is interrupted due to softening and melting of the metal core.
(2) Within the 180–240 A range, the overall duration of insulation smoke emission decreases as the current increases; however, non-monotonic fluctuations occur at medium current levels. Significantly, the ignition time advances with increasing current, indicating that heightened overload currents accelerate the heating and pyrolysis processes of insulation, thereby reducing the ignition delay.
(3) The burning duration exhibits a phased characteristic with changes in current: it increases with a rising current within a lower overload current range, but it decreases and stabilizes at higher currents (≥210 A).
(4) After reaching a relatively high level in the medium current range, the maximum flame height fluctuates within a limited range. The maximum segmented flame width peaks at 200 A and then decreases significantly with increasing current. This indicates that accelerated metal core failure and the rapid evolution of segmented combustion zones at high currents suppress the flame’s lateral expansion.
(5) A multi-indicator fire hazard evaluation system was established based on indicators such as insulation, smoke generation, ignition, combustion persistence, and flame morphology. This system was integrated with the entropy-weighted TOPSIS method for comprehensive quantification. The results indicate that the fire hazard does not increase monotonically with current. Rather, it peaks under moderate overload conditions (200 A). Although ignition occurs earlier at higher currents, sustained combustion and flame propagation are constrained by rapid melting processes, which prevent further escalation of the overall hazard.

In summary, this study elucidates the coupled ignition–combustion–melting evolution of polyethylene-insulated wires under overload current conditions and demonstrates a non-monotonic fire hazard response governed by the competition between intensified Joule heating and accelerated conductor fusing. The proposed multi-indicator evaluation framework provides quantitative support for overload fire-risk stratification. It should be noted that a constant DC overload current was adopted to ensure stable and controllable thermal input, thereby isolating the fundamental thermal–electrical coupling mechanisms. In practical low-voltage systems, AC operation and transient fluctuations may influence failure dynamics; therefore, the present conclusions are most directly applicable to sustained overload scenarios, and further studies under AC and transient conditions are warranted.

## Figures and Tables

**Table 1:** Specifications of wire.

Wire Type	Overall Diameter (mm)	Wire Core Diameter (mm)	Insulation Thickness (mm)	Rated Current (A)	Rated Voltage (kV)
Flame-retardant PE insulated wire	1.78	1.03	0.75	20	0.6/1

**Table 2:** Key Parameters for Ignition and Combustion of Polyethylene Wires under Different Overload Current Conditions.

Current/A	$t_{smoke}$/s	$t_{ign}$/s	$t_{burn}$/s	$H_{\max}$/cm	$W_{\max,seg}$/cm
180	15.30	46.80	16.55	2.34	9.67
190	12.45	41.25	18.80	7.17	14.90
200	11.12	36.82	24.60	5.95	27.94
210	8.25	30.55	19.45	5.54	12.57
220	10.00	27.85	17.15	5.47	18.87
230	7.00	24.65	17.60	6.49	12.42
240	4.80	20.40	17.85	5.99	11.46

**Table 3:** Weights of Indicators Determined by the Entropy Weighting Method.

Indicators	$w_{i}$
t_burn_	0.312
W_max,seg_	0.287
t_ign_	0.155
t_smoke_	0.144
H_max_	0.101

## Data Availability

The raw data supporting the conclusions of this article will be made available by the authors on request.
